# Serum RANKL levels in Chinese patients with ankylosing spondylitis: a meta-analysis

**DOI:** 10.1186/s13018-021-02721-x

**Published:** 2021-10-18

**Authors:** Feifei Ni, Yanchao Zhang, Yi Peng, Xiaoxiao Peng, Jianjun Li

**Affiliations:** 1 Department of Orthopaedics, Shengjing Hospital of China Medical University, Sanhao Street No. 36, Heping District, Shenyang, Liaoning 110004 People’s Republic of China; 2 Department of Orthopedics, Tianjin Baodi Hospital/Baodi Clinical College of Tianjin Medical University, Tianjin, 301800 People’s Republic of China; 3 Department of Urological, Nantong University Danyang Teaching Hospital, Zhenjiang, 212300 People’s Republic of China; 4 Daxing Teaching Hospital of Capital Medical University, Beijing, 102600 People’s Republic of China

**Keywords:** RANKL, Ankylosing spondylitis, Pathogenesis, Meta-analysis, China

## Abstract

**Objective:**

We aimed to determine the association between serum receptor activator of nuclear factor-kappa B ligand (sRANKL) levels and ankylosing spondylitis (AS) in Chinese patients.

**Methods:**

The PubMed, Cochrane Library, Embase, Chinese Biomedical Database, Web of Science, China National Knowledge Infrastructure, VIP, and Wan Fang databases were searched for studies conducted before October 1, 2020, without language restrictions. STATA version 12.0 and Revman version 5.3 were used to analyze the data. The standard mean differences (SMDs) and corresponding 95% confidence intervals (95% CIs) were calculated.

**Results:**

Twelve clinical case–control studies, including 585 patients with AS and 423 healthy controls, were included. The combined SMD for sRANKL suggested that the sRANKL level was significantly higher in Chinese patients with AS than in healthy controls (SMD: 3.27, 95% CI 2.11–4.43, *P* < 0.00001). Serum RANKL-related factor osteoprotegerin (OPG) levels (SMD: 0.86, 95% CI 0.09–1.64, *P* < 0.03) were lower in the Chinese patients with AS than in healthy controls, and the RANKL/OPG ratio (SMD = 1.05, 95% CI 0.64–1.46, *P* < 0.00001) in Chinese patients with AS was approximately the same as that of healthy controls. Subgroup analysis indicated that patients from North and South China had higher sRANKL levels than controls; the sRANKL levels of patients from South China were higher in the subgroup with a Bath Ankylosing Spondylitis Functional Index (BASFI) of > 4 than those of patients in other subgroups. In terms of duration, patients with AS for > 8 years had higher sRANKL levels than health controls. Other subgroup analyses were conducted by region, language, source of control, age, and Bath Ankylosing Spondylitis Disease Activity Index (BASDAI). In these subgroups, the sRANKL levels were significantly higher in the patients with AS than in healthy controls. The BASFI and BASDAI were sources of heterogeneity.

**Conclusions:**

The sRANKL levels are higher in Chinese patients with AS, especially among those from South China. sRANKL levels may be positively correlated with the pathogenesis of AS among Chinese patients.

## Introduction

Ankylosing spondylitis (AS) is a type of inflammatory arthritis, which belongs to the spondyloarthritis family that includes reactive arthritis and psoriatic arthritis [1]. There are a large number of patients with AS worldwide. The prevalence of AS in China is approximately 0.3%, which is approximately 4 million people of China’s population of 1.4 billion people [2]. Hence, AS leads to serious economic burdens on families and the society [3]. Meanwhile, an in-depth understanding of the pathogenesis of AS may address the problems associated with delayed diagnosis of AS and insufficient therapeutic strategy for the disease [4]. Features of AS include the anatomical distribution of the affected joints, types of joint damage, extra-articular manifestations, and sex-related distribution and eyes, intestine, and skin effects [5, 6]. Recent studies have suggested that cytokines, including leptin, adiponectin, and resistin, may play important roles in the pathogenesis of AS [7]. When inflammation occurs, new bone formation leads to bone sclerosis, which can lead to AS; reports have indicated that osteopenia and osteoporosis both occur in AS [8]. Receptor activator of NF-kappa B ligand (RANKL), which was first found on the surface of osteoblasts, also plays an important role in different stages of bone cell metabolism [9, 10]. It is a transmembrane protein that belongs to the tumor necrosis factor (TNF) superfamily [11], which comprises 316 amino acids [12], and is mainly expressed in the bone surface and lymphoid tissue [13]. RANKL and its RANKL receptor play important roles in bone metabolism and the immune system [14]. RANKL adherence to the bone surface is necessary to promote osteoclast differentiation, activation, and survival and accelerates the progress of osteoclast biology [15, 16]. However, osteoclast overactivation leads to bone resorption and has been observed in a variety of bone diseases, such as bone metastasis and osteoporosis; likewise, RANKL is necessary for osteoclast differentiation and immune regulation [17]. Magnetic resonance imaging (MRI) results have indicated that bone inflammation and osteitis are associated with the presence of RANKL [18]. Targeted deletion of RANKL in bone cells prevents osteoclast formation [19].

Recently, several studies have shown that serum RANKL (sRANKL) levels are correlated with AS disease activity and are significantly elevated in patients with AS [20, 21]. However, other studies have found no clear link between RANKL and AS in Asians [22–24]. The relationship between sRANKL and AS among Chinese patients is still unclear. Thus, we performed this meta-analysis to assess the link between sRANKL level and AS in Chinese individuals and to determine which diagnosis and treatment of AS are more convenient and effective.

## Materials and methods

### Literature search

We searched the following electronic databases without any language restrictions: PubMed, Cochrane library, Embase, Chinese Biomedical Database (Chinese database), Web of Science, Chinese National Knowledge Infrastructure (Chinese database), VIP (Chinese database), and Wan Fang (Chinese database). The search strategy was highly sensitive and was performed using a combination of the following keywords and MeSH terms: “Ankylosing Spondylitis” or “Ankylosing Spondylarthritides” or “AS” and “RANKL” or “OPGL Protein” or “Osteoclast Differentiation Factor” or “Osteoprotegerin Ligand” or “TRANCE Protein.”

### Selection criteria

The selection criteria were as follows: (1) only case–control studies in the population to explore the relationship between sRANKL and AS; (2) patients who meet the modified New York criteria or Assessment of SpondyloArthritis international Society [25, 26]; (3) articles should be associated with sRANKL concentration; (4) sufficient and original data; and (5) articles in Chinese should have an English abstract. Studies that did not meet the selection criteria were excluded. If one author published different studies about the same topic, the most recently published or the study with the largest sample size was selected. All studies identified were investigated independently for eligibility by two of the authors (Feifei Ni and Yanchao Zhang), who browsed the title and abstract to select eligible studies. If any reviewer browsed a title or an abstract that met the screening criteria, the full text was browsed.

### Data extraction

From the selected articles, two researchers (Feifei Ni and Xiaoxiao Peng) independently extracted and recorded the required information. Disagreements over data or included studies were resolved through discussion of all items. The recorded information included surname of initial authors, region, language, publication years, age, duration, the Bath Ankylosing Spondylitis Functional Index (BASFI, used to define and to monitor physical functioning in patients with ankylosing spondylitis), the Bath Ankylosing Spondylitis Disease Activity Index (BASDAI, used to measure patient-reported disease activity in patients with ankylosing spondylitis) [27], sRANKL detection method, sRANKL and osteoprotegerin (OPG) levels in the cases and controls.

### Quality of the study

Two researchers (Feifei Ni and Xiaoxiao Peng) used the Newcastle–Ottawa Scale (NOS) to assess the quality of the included studies [28]. The NOS comprises three aspects: (1) subject selection: 0–4; (2) comparability of subject: 0–2; and (3) clinical outcome: 0–3. The NOS scores range from 0 to 9 with two levels of included studies: low quality (0–6) and high quality (7–9). When the two researchers disagreed or when there were discrepancies in the NOS score of a study, a third reviewer intervened.

### Statistical analyses

The relationship between sRANKL levels and AS susceptibility was assessed using the standardized mean differences (SMDs) and 95% confidence intervals (95% CIs). Cochran’s Q-statistic (*P* < 0.05 was considered significant) and *I*^2^ tests were used to quantify heterogeneity among studies [29]. The random effects model was used when heterogeneity was significant (*P* < 0.05 for the Q test or *I*^2^ test exhibited > 50%); otherwise, the fixed-effects models were used [30]. When heterogeneity was significant, subgroup analysis was performed to find the potential reasons for the difference in sRANKL levels between patients with AS and healthy controls. In addition, sensitivity analysis was used to assess if a single study had an impact on the whole assessment. The impact of publication bias was analyzed using Egger’s test (*P* < 0.05 was considered significant), which can be used to evaluate the funnel plot asymmetry that reveals potential publication bias [31] [32]. The data were analyzed using the software Review Manager 5.3 and STATA version 12.0.

## Results

### Inclusion criteria

**Fig. 1:**
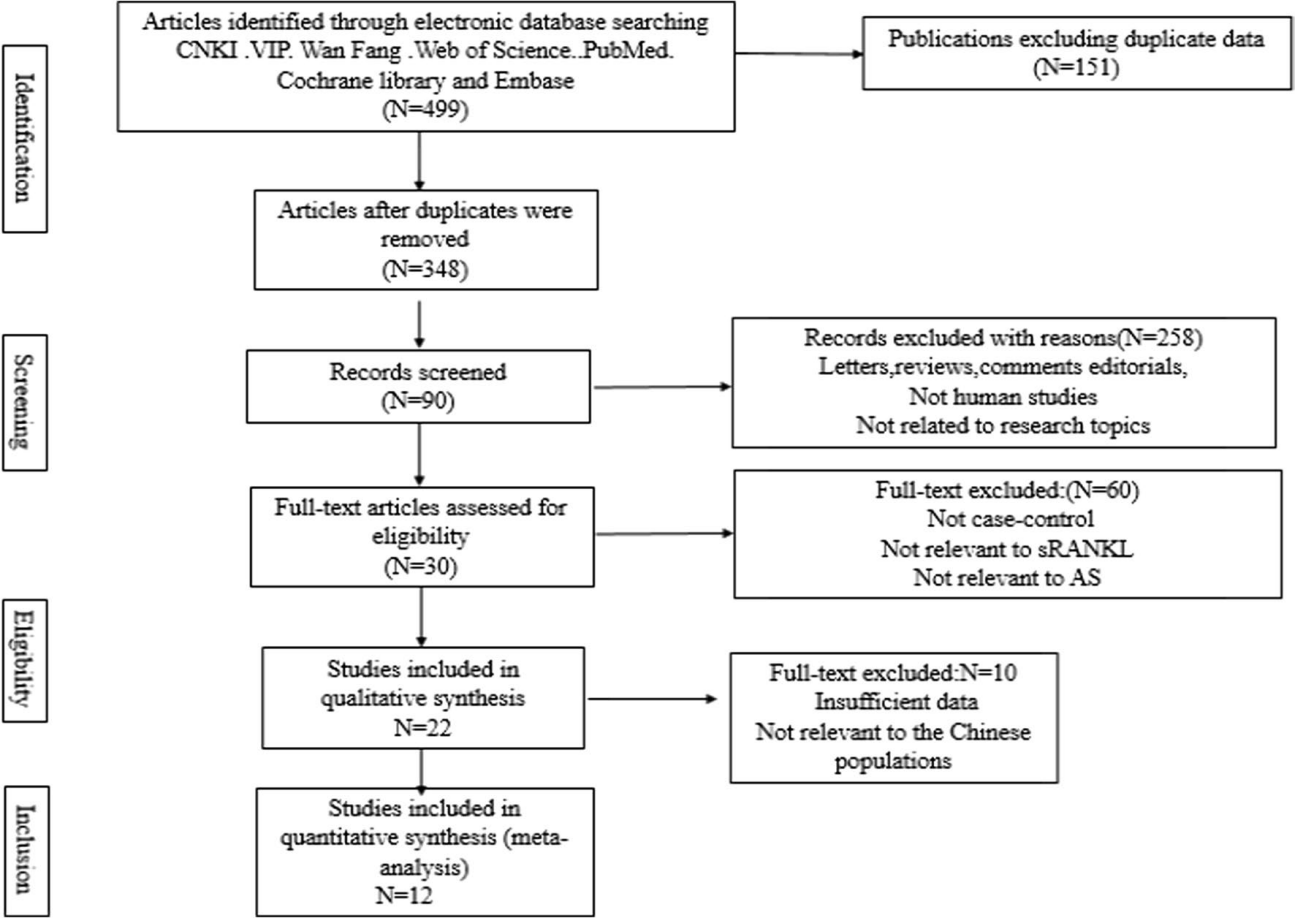
Study selection flow chart

We selected 499 potentially relevant articles from eight databases. After deleting duplicates, 348 records remained. By skimming the titles and abstracts, we excluded 258 papers due to at least one of following reasons: (1) 6 articles were comments, 5 were letters, 25 were reviews, and 9 were editorials; (2) 156 were not related to the research topics; and (3) 57 were not on human studies. Full-text articles from the remaining 90 articles were reviewed again and 60 trials were excluded (30 were not case–control studies, 18 were not relevant to RANKL, and 12 were not relevant to AS), leaving 30 studies to the next selection step. After studies that were not related to the Chinese population and those lacking data integrity were removed, 12 studies were finally selected [33–44] (Fig. 1).

### Features of the studies

Twelve studies, comprising 585 patients with AS and 423 controls, were included in accordance with the selection criteria. The basic features of the studies are shown in Table 1. sRANKL levels in all 12 studies were tested using enzyme-linked immunosorbent assay. The methodological quality assessment using the NOS is shown in Table 1.

**Table 1:** Characteristics of included studies

Author	Year	Region	Language	Study type	Criteria for disease	Case	Control	Case	Control	Case	Control	Disease durations	Source for control	Method	NOS
An et at.	2010	Hebei	Chinese	Case–control	New York (1984)	30	20	30/0	20/0	28 ± 8	30 ± 5	7 ± 7	HB	Elisa	7
Shen et al.	2019	Liaoning	Chinese	Case–control	New York (1984)	202	98	102/100	49/49	43.4 (24–65)	44.8 (22–65)	–	HB	Elisa	7
Chen et al.	2010	Taiwan	English	Case–control	New York (1984)	42	26	38/4	–	33.6 ± 12.14	–	8.17 ± 8.2	NA	Elisa	7
Hou et al.	2018	Shandong	English	Case–control	New York (1984)	40	40	31/9	31/9	31.7 ± 2.1	31.4 ± 2.5	7.4 ± 1.9	PB	Elisa	7
Li et al.	2013	Guangdong	Chinese	Case–control	New York (1984)	44	15	32/12	9/6	31 ± 0.7	29 ± 0.4	–	HB	Elisa	7
Luo et al.	2011	Jiangsu	Chinese	Case–control	New York (1984)	44	44	23/21	22/22	42.2 ± 11.9	4. ± 11.1	7.3 ± 4.8	HB	Elisa	7
Wei et al.	2013	Guangdong	Chinese	Case–control	New York (1984)	40	40	40/0	40/0	33.67 ± 6.88	30.95 ± 6.04	12.63 ± 9.68	NA	Elisa	7
Zhang et al.	2018	Hebei	Chinese	Case–control	New York (1984)	46	38	30/16	28/10	27.2 ± 8	25.26 ± 8.67	7.6 ± 3.8	HB	Elisa	7
Zhao et al.	2010	Fujian	Chinese	Case–control	New York (1984)	23	17	16/7	9/8	33 ± 14	52 ± 14	–	PB	Elisa	7
Huang et al.	2018	Zhejiang	Chinese	Case–control	New York (1984)	21	42	6/15	15/26	44.48 ± 19.77	46.24 ± 17.79	–	HB	Elisa	7
Zhang et al.	2015	Guangdong	Chinese	Case–control	ASAS	21	21	18/4	17/5	31 ± 9	30 ± 9	6 ± 3	HB	Elisa	7
Zhang et al.	2020	Guangdong	English	Case–control	ASAS	32	22	25/7	18/4	28.5 (15–26)	30.5 (18–56)	4.68 ± 3.63	PB	Elisa	7
		Province										Years			

M = male, F = female. NOS = Newcastle–Ottawa Scale, HB = hospital based, PB = population based

### Meta-analysis in AS

**Fig. 2:**
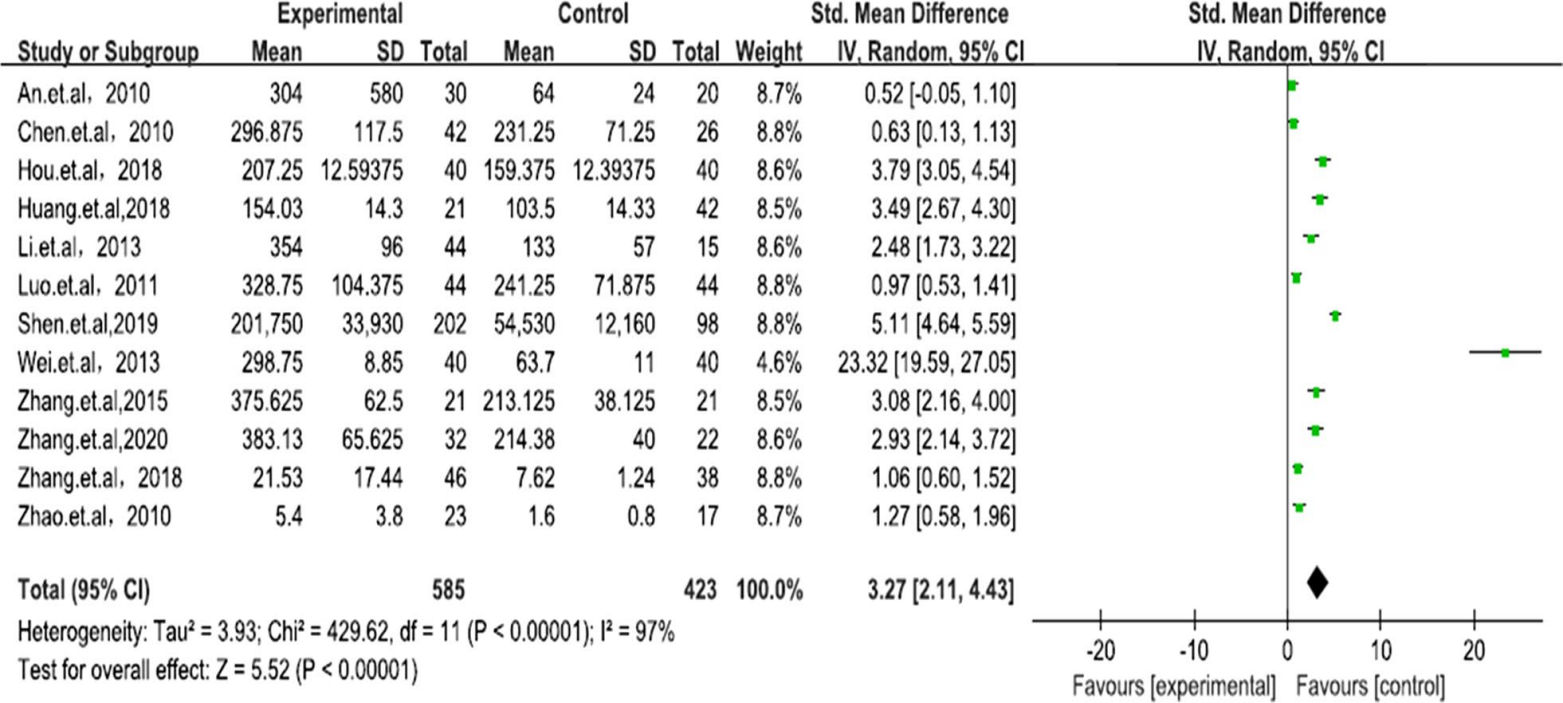
Forest plot of sRANKL: patients with AS versus controls

**Fig. 3:**
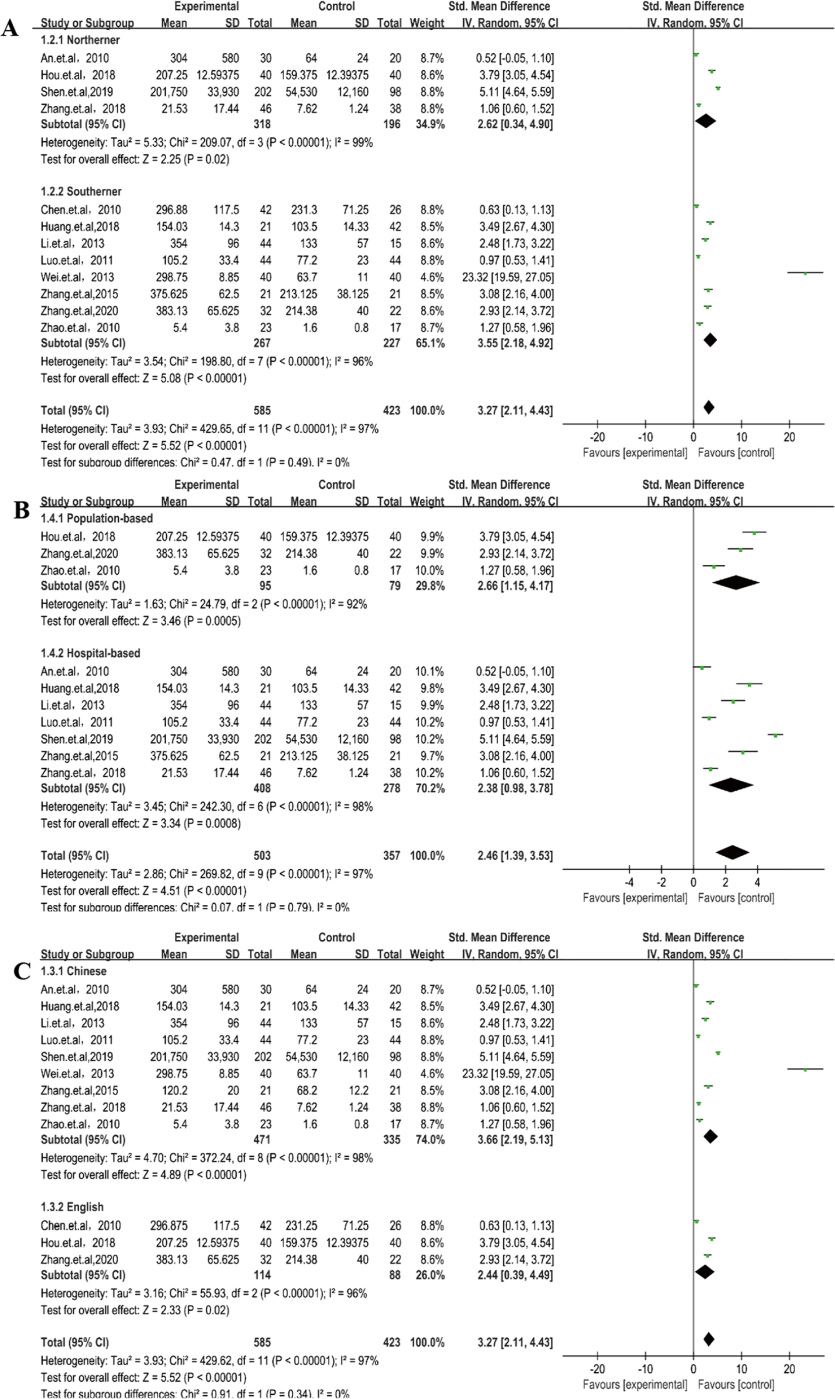
sRANKL level, language, source of control, and ethnicity: cases versus controls

**Fig. 4:**
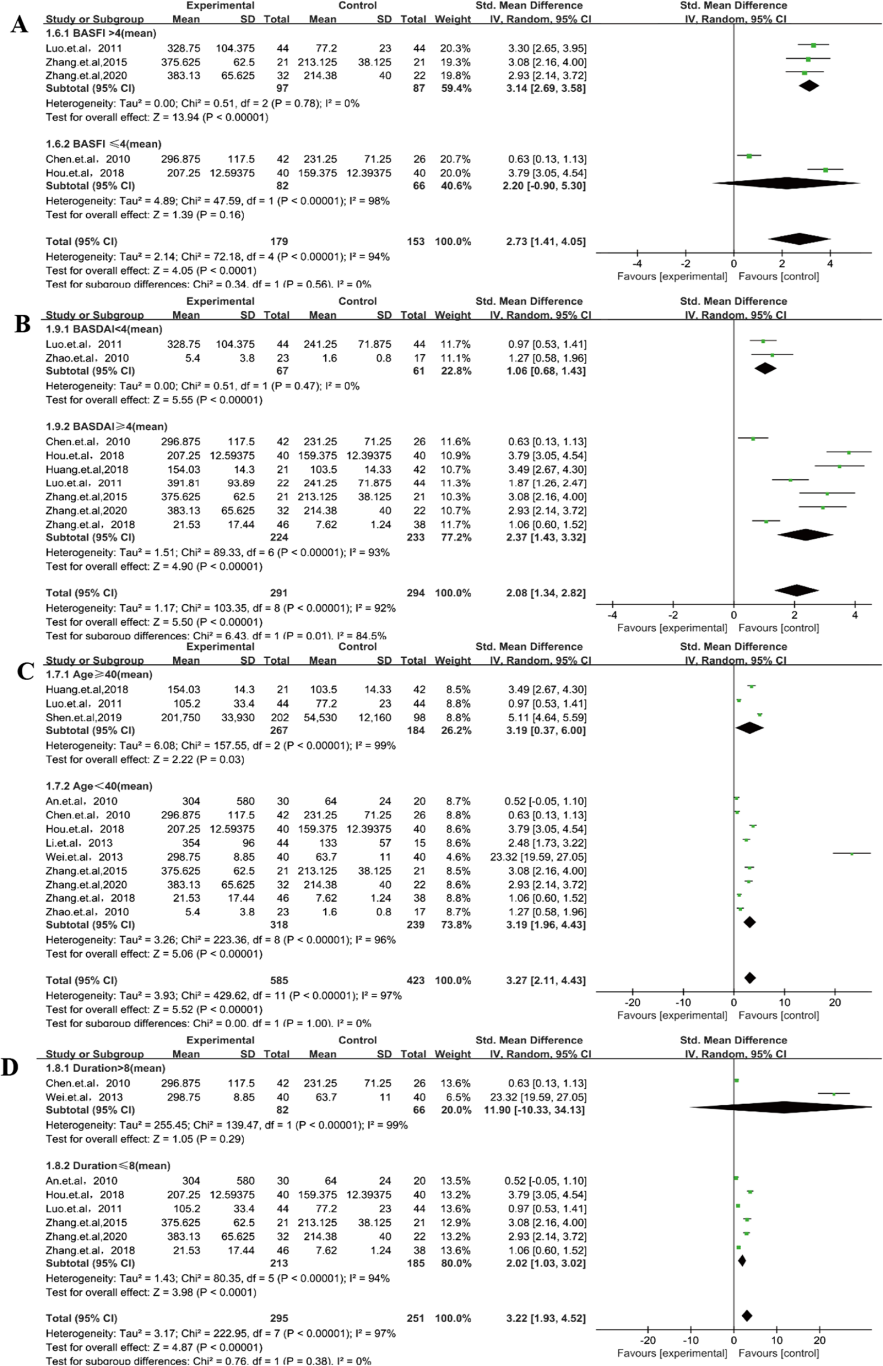
sRANKL level, the BASDAI, the BASFI, age, and duration: cases versus controls

Significant heterogeneity was found in the 12 studies (*P* < 0.00001, *I*^2^ = 97%), and the random-effects model showed that the sRANKL levels in patients with AS were statistically different compared with those in controls (SMD = 3.27, 95% CI 2.11–4.43, *P* < 0.00001) (Fig. 2). Subgroups, including language, source of control, age, BASFI and the BASDI, were analyzed. In terms of these subgroups, the sRANKL levels of patients with AS were significantly higher than those of controls (Figs. 3, 4).

**Fig. 5:**
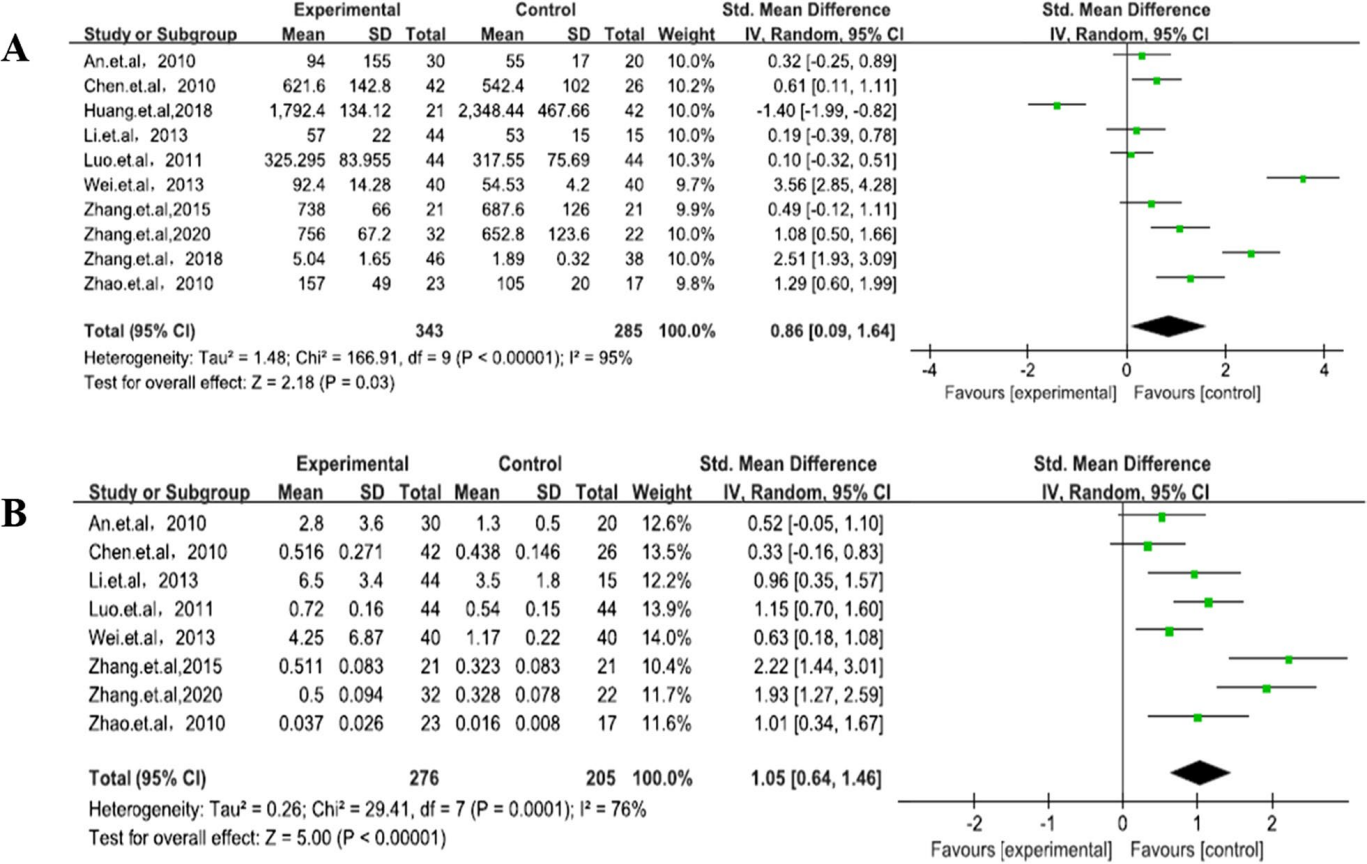
sOPG levels and RANKL/OPG ratio: cases versus controls

Subgroup analysis by region was divided into patients from North and South China because of the geographical differences in China’s population distribution. Patients with AS in the two groups obviously had higher sRANKL levels than controls, although the patients from the south had higher sRANKL levels (SMD = 3.55, 95% CI 2.18–4.92, *P* < 0.00001) than those of patients from the North (SMD = 2.62, 95% CI 0.34–4.9, *P* < 0.02). Further subgroup analysis indicated that a BASFI of > 4 (SMD = 3.14, 95% CI 2.69–3.58, *P* < 0.00001) and duration of ≤ 8 years (SMD = 2.02, 95% CI 1.03–3.02, *P* < 0.0001) had a positive correlation in patients with AS, although a BASFI of ≤ 4 (SMD = 2.2, 95% CI − 0.9 to 5.3, *P* = 0.16) and duration of > 8 years (SMD = 11.9, 95% CI − 10.33 to 34.13, *P* = 0.29) did not (Figs. 3, 4). sRANKL-related factor serum OPG levels (SMD = 0.86, 95% CI 0.09–1.64, *P* = 0.03) (Fig. 5) in patients with AS were lower than those in controls, and the RANKL/OPG ratio (SMD = 1.05, 95% CI 0.64–1.46, *P* < 0.00001) (Fig. 5) in patients with AS was higher than that in controls. All results showed that high expression of sRANKL was an important risk factor for the occurrence of AS in the Chinese population.

### Sensitivity analysis and publication bias

**Fig. 6:**
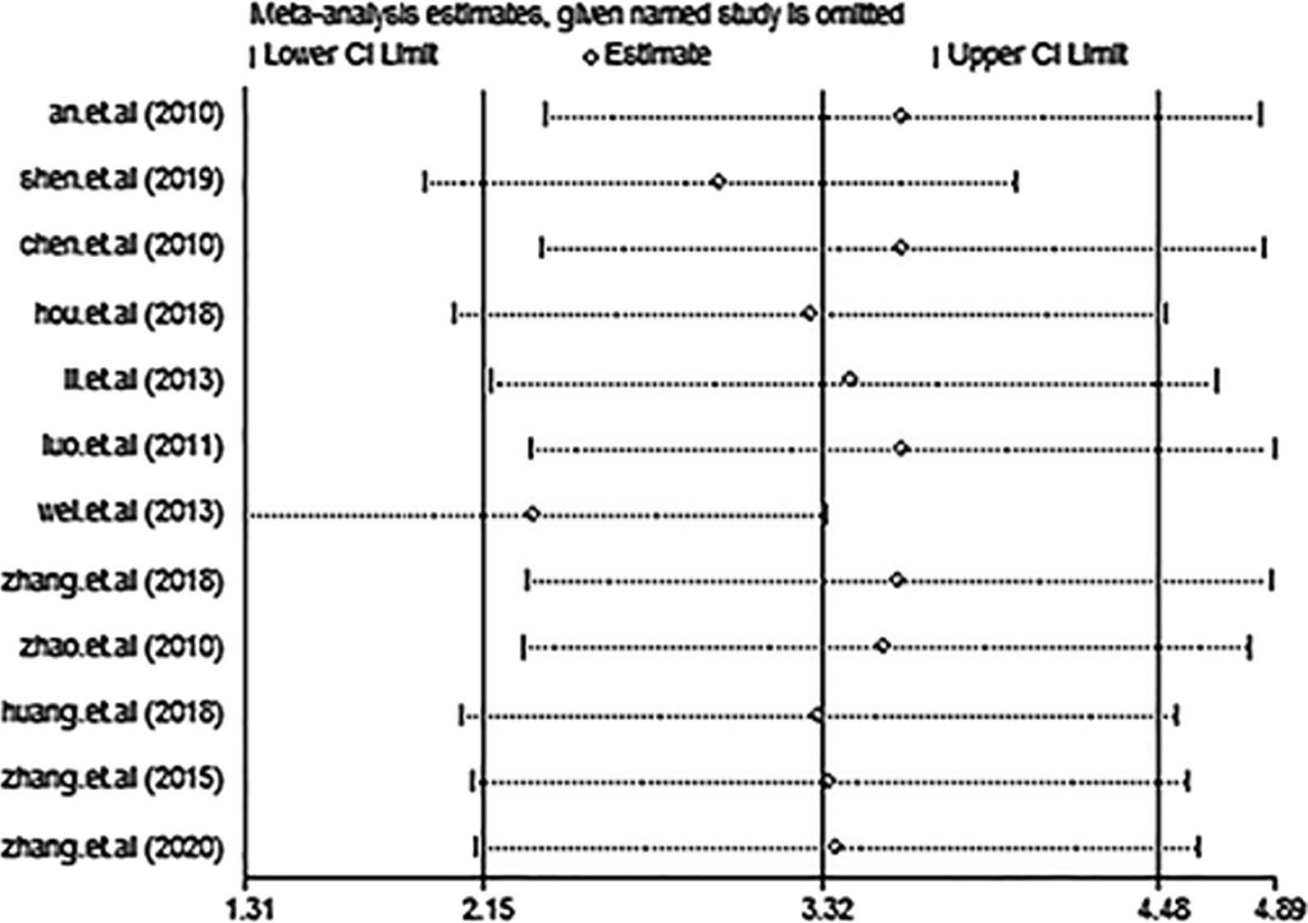
Forest plot in the sensitivity analysis. CI: confidence interval

**Fig. 7:**
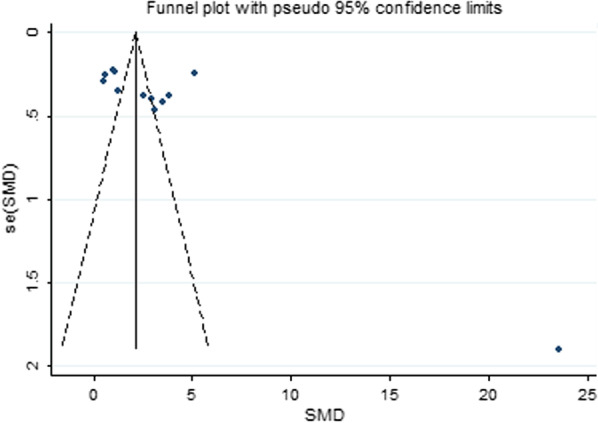
Shape of the funnel plot. Egger’s test, *t* = 2.16, *P* = 0.056

The results of the sensitivity analysis indicated that none of the studies had an effect on the overall estimate of the association between RANKL levels and AS risk. Thus, the data presented in our meta-analysis were relatively stable and credible (Fig. 6). The graphical funnel plots of the 12 included studies were symmetrical, and Egger’s test showed no publication bias (*P* = 0.056) (Fig. 7).

## Discussion

In this study, we evaluated the sRANKL levels in Chinese patients with AS from 12 articles through meta-analysis, and we investigated the probable relationship between sRANKL level and AS. Our results suggested that RANKL may play a key role in the pathogenesis of AS in Chinese patients. AS is a chronic, progressive systemic rheumatism that affects the sacroiliac joints, central axis bones, peripheral joints, and other extra-articular organs [45]. A recent study suggests that AS pathogenesis involves bone resorption and formation [46]. Although many studies have evaluated the correlation between sRANKL and AS in the Chinese population, their results are controversial [47–49]. The specific number of subjects in the control group of the 47th citation was unclear. The 48th citation does not have RANKL specific data. The 49th citation is a self-control study. Formal requests were made through e-mails to the authors of some studies (the 47th and 48th citations) to obtain their data, although no response was received. Therefore, in line with our screening criteria, these three articles were finally removed. Therefore, we conducted this study to investigate the association between sRANKL and AS. In the process of AS-associated peripheral joint ossification, coupling imbalance between osteoblasts and osteoclasts is a condition that cannot be neglected with regard to ossification [50]. RANKL produced by osteocytes plays an important role in osteoclast formation and bone reconstruction [51]. The RANKL/RANK/OPG pathway controls osteoclastic activity and formation and plays an important role in the pathogenesis of AS [52]. Denosumab is a monoclonal antibody against RANKL that prevents osteoclast formation and has been used as a first-line treatment for osteoporosis. [53] In a study of patients with active Rheumatoid arthritis (RA) 6–12 months of denosumab therapy led to a successful reduction in radiological progress, improved bone mineral density (BMD) of the lumbar spine and entire hip, and significantly reduced bone erosion, along with reduction in bone turnover markers [54]. Another study showed that patients with RA who received 180 mg of denosumab had reduced joint erosion according to MRI measurement at 6 months after treatment [55]. In addition, studies have reported that denosumab treatment can significantly reduce the loss of BMD around the prosthesis after total knee arthroplasty. This treatment strategy can promote early stable fixation of the prosthesis [20]. A study also showed that using tripterygium to reduce the expression of RANKL in the blood can significantly reduce the disease activity in patients with AS [20]. Therefore, lowering the RANKL level in serum may be beneficial to the progression of AS. OPG in bone formation is a kind of protective factor conducive to the growth of osteoblasts, normal osteoblasts, and osteoclasts in the body in a dynamic balance with RANKL; both are not excessively activated but otherwise may lead to bone disease [56]. The RANKL/OPG ratio is closely related to osteoclast formation and maturity. In this study, the OPG level in peripheral blood of patients with AS (SMD = 0.86, 95% CI 0.09–1.64, *P* < 0.03) was significantly lower than of healthy controls, although the sRANKL level (SMD = 3.27, 95% CI 2.11–4.43, *P* < 0.00001) was significantly higher in patients with AS than in healthy controls. As such, osteoblastic activity was restrained. In-depth studies have shown that the RANKL/OPG ratio determines the direction of bone change. As the ratio decreases, bone loss decreases [57]. In this study, the RANKL/OPG (SMD = 1.05, 95% CI 0.64–1.46, *P* < 0.00001) ratio was higher in Chinese patients with AS than in controls. This result suggests that excessive activation of osteoclasts increases inflammation as confirmed in animal models. OPG gene knockout mice had insufficient osteoblast production and decreased bone mass, and this leads to severe osteoporosis with a high incidence of bone fracture [58]. The RANKL knockout mice developed severe osteosclerosis, and only a small number of osteoclasts were observed in the bone tissue of those mice [59, 60]. In addition, RANKL-positive osteocytes were elevated in animal models of inflammation, such as periodontitis and spinal injuries [61]. Osteoblasts and activated T cells also produce RANKL to regulate adaptive immunity [12, 62]. Some studies have found that RANKL is expressed on the surface of T cells and lymphocytes, and this regulates lymph node formation and T cell and dendritic cell communication. Overactivation of the immune system may contribute to the disease process of AS [63]. RANKL-expressed T cells can affect osteoclast formation, which explains bone loss in patients with chronic inflammatory diseases [16]. Recently, CD4 + T and CD8 + T cells were confirmed to participate in the pathogenesis of AS, although many problems are yet to be resolved. Furthermore, RANKL overexpression in T cells in RANKL knockout mice can restore osteoclast production and lead to a partial return of the normal bone marrow cavity [64]. Meanwhile, in RANKL deficient mutant mice, the lack of osteoclasts leads to severe osteoporosis and failure of tooth and lymph node formation [65]. Thus, bone loss due to inflammation may arise from the complex interactions of bone cells, T and B cells, and signaling pathways, such as the RANKL/RANK/OPG pathway [66]. This phenomenon may explain the roles of systemic activation of T cells and RANKL production through T cells as important mediators of bone loss in vivo [67]. In autoimmune diseases, arthritis, or local inflammation of the bone caused by infections, T cells are usually activated first, leading to the overexpression of RANKL and consequent bone loss [68].

Considering that other related factors may have a connection with high levels of sRANKL and AS pathogenesis, a stratified analysis based on region, language, source of control, age, duration, the BASDAI, and the BASFI, was conducted. The BASDAI and BASFI were representative of the activity of the disease [27]. In the current study, sRANKL in the disease group of BASDAI > 4 and BASFI > 4 was significantly higher than that in the control group. sRANKL in the subgroup with a disease duration of > 8 years was significantly higher than that in the control group and may be attributed to the abnormal activation of the immune system and inflammatory cytokine in early AS [69]. The longer the course of the disease, the higher the activity of the disease and the more obvious are the systemic symptoms. It was found in studies with an average course of > 8 years that the longer the duration of AS, the greater the risk of cardiovascular disease, hearing loss, and poor physical mobility [70, 71]. At the same time, AS causes the lung interstitium to develop lesions at an early stage, and the range of lesions increases with the duration of the disease [72]. In this meta-analysis, we observed similar results. Regardless of age ≥ 40 years or < 40 years in the subgroup, the serum RANKL level of patients with AS was significantly higher than that of controls, indicating that age may be an influencing factor on serum RANKL levels in patients with AS. The BASDAI is an indicator for assessing disease activity, and a score of > 4 indicates that the disease is in the active phase. The BASFI is a recognized standard for assessing the functional ability of patients with AS; the larger the score, the worse the spinal function [73]. Many research reports show that anti-inflammatory treatment can significantly improve the BASDAI and BASFI, and this could also lead to significant reduction in RANKL levels [74, 75]. Therefore, we speculate that serum RANKL levels may have a potential role in the assessment of inflammation and functional status of patients with AS. All these findings suggest that a BASDAI of > 4, a BASFI of > 4, and the duration of the disease affect sRANKL expression. We also found that subgroup BASDAI < 4 and BASFI > 4 are sources of heterogeneity. In terms of region, the relationship was significant among Chinese patients with AS, especially among those patients from the South. This result may be attributed to differences in China’s vast territory and geographical distribution of people, genetics, diet, and living environment. Studies have reported that there is a significant delay in diagnosing patients with AS in the south compared with those in the north. This may be due to the large number of mountainous areas in Southern China and inconvenient transportation, which is not conducive for patient examination and treatment, and may lead to the progression of the disease. However, there are many plains in the north and transportation is convenient, which makes it conducive to diagnose and to treat such patients [76]. In fact, HLA-B*2704 is the main subtype of the Chinese population, while HLA-B*2704 is the main subtype of patients with AS, and its carriers are more in South China, compared with those in the north [77]. Some studies have shown that a low starch diet can reduce inflammation and symptoms in patients with AS [78], and a high-fat diet is positively correlated with BASDAI activity. Long-term exposure to PM2.5 is closely related to the BASMI, BASFI, and BASDAI scores of patients with AS. Furthermore Long-term exposure to pollution can cause severe symptoms in patients with AS [79]. Therefore, more in-depth research is needed to explore the pathogenesis of AS in Chinese patients in the future.

This meta-analysis has some limitations. First, the small sample size of the 12 studies may have affected our results. Second, articles that provide only medians and ranges or upper quartiles and lower quartiles were excluded because if we converted these data, the result of the transformation may not have been accurate even if a method of transformation had been reported by Hozo et al. [24, 80, 81]. Third, in the 12 studies, information on factors that affect sRANKL, such as HLA-B27, body mass index, and sex, were not detailed enough. Therefore, we cannot safely further analyze the relationship between serum RANKL and AS given that the sex ratio between male and female individuals may have an impact on the reliability of our study. Despite the above limitations, this is the first meta-analysis on the association between sRANKL levels in Chinese patients with AS and those in healthy controls.

In conclusion, our study indicated that sRANKL levels in Chinese patients with AS especially in patients with AS in the south, were obviously higher than those in the healthy controls. sRANKL level may have a positive correlation with the pathogenesis of Chinese patients with AS and could serve as a promising biomarker for the severity of AS in Chinese patients. The results of our study may ultimately contribute to the development of new treatments methods for bone damage in Chinese patients with AS, as this field has not been thoroughly studied. Further intensive study on Chinese individuals with a large sample size are needed.

## Conclusions

The findings of our study suggests that sRANKL has a positive correlation with the pathogenesis of AS in Chinese patients and may potentially serve as a biomarker for the severity of AS in Chinese populations.

## Data Availability

The authors confirm that the data supporting the findings of this study are available within the article.

## Abbreviations

AS: Ankylosing Spondylitis
RANKL: Receptor activator of nuclear factor-kappa B ligand
OPG: Osteoprotegerin
TNF: Tumor Necrosis Factor
BASFI: Bath Ankylosing Spondylitis Functional Index
BASDAI: Bath Ankylosing Spondylitis Disease Activity Index
CI: Confidence interval
SMD: Standard mean difference
M: Male
F: Female
RA: Rheumatoid arthritis
NOS: Newcastle–Ottawa Scale
HB: Hospital based
PB: Population based
PRISMA: Preferred Reporting Items for Systematic Reviews and Meta-Analyses
ELISA: Enzyme-linked immunosorbent assay
